# Time-Dependent Unitary Transformation Method in the Strong-Field-Ionization Regime with the Kramers-Henneberger Picture

**DOI:** 10.3390/ijms22168514

**Published:** 2021-08-07

**Authors:** Je-Hoi Mun, Hirofumi Sakai, Dong-Eon Kim

**Affiliations:** 1 Department of Physics and Center for Attosecond Science and Technology, POSTECH, Pohang 37673, Korea; kimd@postech.ac.kr; 2 Max Planck POSTECH/KOREA Research Initiative, Pohang 37673, Korea; 3 Department of Physics, Graduate School of Science, The University of Tokyo, 7-3-1 Hongo, Bunkyo-ku, Tokyo 113-0033, Japan; hsakai@phys.s.u-tokyo.ac.jp; 4 Institute for Photon Science and Technology, Graduate School of Science, The University of Tokyo, 7-3-1 Hongo, Bunkyo-ku, Tokyo 113-0033, Japan

**Keywords:** numerical method, laser-matter interaction, time-dependent Schrödinger equation, time-dependent unitary transformation method, strong-field ionization, Kramers-Henneberger frame

## Abstract

Time evolution operators of a strongly ionizing medium are calculated by a time-dependent unitary transformation (TDUT) method. The TDUT method has been employed in a quantum mechanical system composed of discrete states. This method is especially helpful for solving molecular rotational dynamics in quasi-adiabatic regimes because the strict unitary nature of the propagation operator allows us to set the temporal step size to large; a tight limitation on the temporal step size (δt<<1) can be circumvented by the strict unitary nature. On the other hand, in a strongly ionizing system where the Hamiltonian is not Hermitian, the same approach cannot be directly applied because it is demanding to define a set of field-dressed eigenstates. In this study, the TDUT method was applied to the ionizing regime using the Kramers-Henneberger frame, in which the strong-field-dressed discrete eigenstates are given by the field-free discrete eigenstates in a moving frame. Although the present work verifies the method for a one-dimensional atom as a prototype, the method can be applied to three-dimensional atoms, and molecules exposed to strong laser fields.

## 1. Introduction

Over the last few decades, the ionization of atoms and molecules by ultrafast strong infrared laser fields has attracted considerable interest because of the availability of high-intensity lasers. Field-induced ionization can be divided into two regimes, according to the Keldysh parameter $\gamma \equiv \sqrt{I_{P}/\left(2U_{P}\right)}$ [1], where $I_{P}$ and $U_{P}$ are the ionization and the ponderomotive potentials, respectively. The ionization dynamics is considered to be governed by tunneling when γ<<1, while for γ>>1 the process is mostly affected by multiphoton ionization. For a typical 800-nm infrared laser field, the dynamics of the atoms and molecules can be characterized by the laser intensity. In the low-intensity laser field (γ>>1), the dynamics can be studied with the aid of the perturbation theory. When the laser field strength is comparable to or even higher than the Coulomb field strength in atoms and molecules (γ<<1), the ionization dynamics can be described by tunneling ionization, which can be solved analytically by using a strong-field approximation (SFA) model [1,2]. The tunneling picture based on the SFA model explains many qualitative features of strong-field phenomena, such as high-harmonic generation (HHG) [3,4,5,6] and above-threshold ionization (ATI) [4,7,8].

However, the conventional SFA model is not capable to render a description of phenomena mediated by other bound states in addition to the ground state. For example, some tunneling-ionized electrons that receive relatively low drift energies from the laser fields are observed to still stay in the excited bound states after the laser pulse has passed [9]. A simple man model, which is based on the solutions of the Newtonian equations of motion, has been used in many studies on this phenomenon known as the frustrated tunneling ionization (FTI) [9,10,11,12,13,14]. Moreover, coherent EUV generation via FTI [15,16] and resonantly enhanced HHG [17,18,19,20,21] require the high-lying electronic bound states to be considered. In solving the full TDSE to understand these phenomena, discrete-level-based calculations and/or analysis are essential. The discrete-level-based analysis of the strong-field ionization can provide fresh insights into the underlying physical mechanisms. In Ref. [22], by calculating a strong-field-dressed discrete adiabatic basis set, it has been revealed that tunneling ionization is diabatic rather than adiabatic in a language based on the so-called adiabatic representation. Tunneling ionization is often regarded as an adiabatic process, which is not true in terms of the adiabatic representation [22]. Furthermore, a series of discrete-level-based numerical calculations have shown that atomic ionization passages can be manipulated by chirp control of an incident laser pulse [23].

Analytical and numerical studies with discrete basis sets are commonly used in various branches of atomic and molecular physics, such as the Rydberg atoms [24,25], ultracold gases and trapped ions [26,27,28], and molecular rotational dynamics [29,30,31]. In molecular rotational dynamics, we have developed a time-dependent unitary transformation (TDUT), which has been particularly useful in quasi-adiabatic regimes. In this method, the field-dressed eigenstates and eigenenergies are calculated in every temporal step to obtain strict unitary propagation operators. The TDUT method is free from a tight limitation on the temporal step size (δt<<1), existing in conventional numerical methods (e.g., Crank–Nicolson method and Runge–Kutta method), so that rapid numerical calculations are possible. In the case of strong-field-induced ionization dynamics, however, specific efforts are needed to apply this approach using discrete field-dressed states as a basis set. The eigenstates of an atomic potential tilted by a strong electric field form continuum states [22], and the ground state is localized at the edge of a spatial boundary position. Thus, the direct formulation of the TDUT method itself [29] cannot solve the dynamics in the presence of ionization events.

In this study, this problem was solved using the Kramers-Henneberger (KH) frame [32], in which the strong-field-dressed discrete eigenstates are given by the field-free discrete eigenstates in a moving frame. Once a unitary translation operator is calculated correctly, the TDSE can be solved numerically using the TDUT method in the KH frame. Using a one-dimensional atom as a prototype, the numerical method is validated by comparing it with the Crank–Nicolson method. The final electronic states excited by a few-optical cycled near-infrared (800 nm) laser are calculated by changing the field strength and initial state. This study numerically clarifies that the number of discrete states for the TDUT calculation depends on the field strength. Below the tunneling intensity regime, only a few bound states can be used, while in the stronger field regime, much higher-lying continuum states need to be included. The dynamics of three-dimensional atoms and molecules can be calculated using the TDUT method.

The paper is organized as follows. Section 2, revisits the general TDUT method in the ionization-free regime, which was developed for the molecular rotational dynamics [29]. The method is then implemented to a strong-field ionization regime within the KH frame. Section 3 presents the numerical results of a one-dimensional atom exposed to an intense laser pulse to test the numerical method. A summary and outlook are provided in Section 4. Atomic units are used throughout the paper unless otherwise stated.

## 2. Numerical Method

This section provides a detailed explanation of the numerical method.

### 2.1. Time-Dependent Unitary Transformation Method (TDUT) in Discretized Systems

In conventional methods for solving the TDSE, a wave function at t+δt, ψ(t+δt), is approximated by $\left[\exp\left(-i\hat{H}\left(t\right)\delta t\right)\right]\psi \left(t\right)∼\left[1-i\hat{H}\left(t\right)\delta t\right]\psi \left(t\right)$ under the assumption of δt≪1. Here, $\hat{H}\left(t\right)$ is the time-dependent Hamiltonian. In this simple sum formulation, the operator $\left[1-i\hat{H}\left(t\right)\delta t\right]$ is not a unitary one in principle. The non-unitary nature is accumulated over a number of evolution steps, and the simulation can catastrophically fail. The popular Runge–Kutta method and Crank–Nicolson method can reduce the non-unitary nature. On the other hand, the constraint δt≪1 must be satisfied to a reasonable degree.

Several numerical methods, such as methods based on the Chebyshev propagator [33] and Lanczos propagator [34], the split-operator method [35], and a huge number of variations, are available. The time-dependent unitary transformation (TDUT) method is one of the effective and intuitive methods for the TDSE [29], where every temporal evolution operator is strictly unitary. To describe the method, we need to consider two arbitrary frames labeled by (a) and (b), respectively. When we consider a single stepwise temporal variation δt, the time-dependent Hamiltonian evolves from $\hat{H}\left(t\right)$ to $\hat{H}\left(t+\delta t\right)$. The two Hamiltonians can be labeled as $\hat{H}\left(t\right)\equiv {\hat{H}}^{a}$ and $\hat{H}\left(t+\delta t\right)\equiv {\hat{H}}^{b}$. Because the time-independent Hamiltonian operator ${\hat{H}}^{a}$ or ${\hat{H}}^{b}$ is a Hermite operator in general, there are a set of real-valued eigenvalues $\epsilon_{i}^{a}$ ($\epsilon_{i}^{b}$) and the corresponding set of eigenstates $\phi_{i}^{a}$ ($\phi_{i}^{b}$), obtained by solving the TISE as follows.

(1) $$\begin{array}{c} {\hat{H}}^{a}\phi_{i}^{a}=\epsilon_{i}^{a}\phi_{i}^{a},\\ {\hat{H}}^{b}\phi_{i}^{b}=\epsilon_{i}^{b}\phi_{i}^{b}. \end{array}$$

Here the integer *i* represents a state number.

Let us set the moment of the step-wise change of the Hamiltonian from (a) to (b) at $t_{0}$. A wave function $\psi (t_{0})$ can be expressed as a superposition of eigenstates $\phi_{i}^{a}$ as follows.

(2) $$\begin{array}{c} \psi \left(t_{0}\right)=\sum_{m}c_{m}^{a}\left(t_{0}\right)\phi_{m}^{a}\left(t_{0}\right), \end{array}$$

where $c_{m}^{a}\left(t_{0}\right)$ corresponds to the probability amplitude $\langle \phi_{m}^{a}|\psi \left(t_{0}\right)\rangle$ at time $t_{0}$. The same eigenstate expansion is also possible in the (b) frame. The probability amplitude $c_{m}^{b}\left(t_{0}\right)\equiv \langle \phi_{m}^{b}|\psi \left(t_{0}\right)\rangle$ in the (b) representation, can be expressed using the (a) frame eigenstates as $c_{n}^{b}\left(t_{0}\right)=\sum_{m}\langle \phi_{n}^{b}|\phi_{m}^{a}\rangle c_{m}^{a}\left(t_{0}\right)$. In the (b) frame, for $t\geq t_{0}$ until the next step-wise change of external field intensity occurs, the time evolution of the wave function is given by adding a phase shift to each eigenstate of $\phi_{n}^{b}$, which is operated by

(3) $$\begin{array}{c} \psi \left(t\right)=\sum_{n}\left[\sum_{m}\langle \phi_{n}^{b}|\phi_{m}^{a}\rangle c_{m}^{a}\left(t_{0}\right)\right]\phi_{n}^{b}\times \\ \exp(-i\epsilon_{n}^{b}\left(t-t_{0}\right)). \end{array}$$

Here, the matrix $\langle \phi_{n}^{b}|\phi_{m}^{a}\rangle$ is equivalent to $\sum_{l}\langle \phi_{n}^{b}|\chi_{l}\rangle \langle \chi_{l}|\phi_{m}^{a}\rangle$, with $\chi_{l}$ being a field-free eigenstate, i.e., the spherical harmonics in the case of the linear molecules. The unitary matrices $\langle \phi_{m}^{a}|\chi_{l}\rangle$ and $\langle \phi_{m}^{b}|\chi_{l}\rangle$ are obtained by solving the TISE given in Equation (1). In a matrix-based expression, the temporal evolution can be rewritten by an evolution matrix, ${\hat{U}}_{t_{0}}$, satisfying $\hat{\psi }\left(t_{0}+\delta t\right)\equiv {\hat{U}}_{t_{0}}\hat{\psi }\left(t_{0}\right)$, as follows:

(4) $$\begin{array}{c} {\hat{U}}_{t_{0}}=\exp\left(-i{\hat{\epsilon }}^{b}\delta t\right){\hat{U}}^{b}{\hat{U}}^{†a}. \end{array}$$

**Figure 1:**
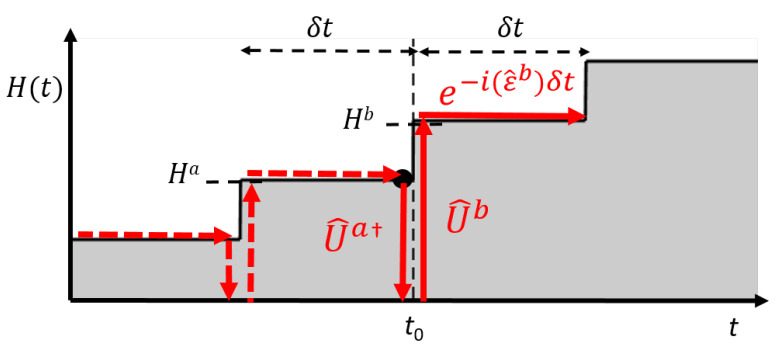
Schematic diagram of time-dependent unitary transformation method for wave packet evolution. In the presence of the time-dependent Hamiltonian $\hat{H}\left(t\right)$, temporal evolution of a wave function from $t=t_{0}$ to $t=t_{0}+\delta t$ is operated by multiplying three strict unitary operators ${\hat{U}}^{†a}$, ${\hat{U}}^{b}$, and $\exp(-i{\hat{\epsilon }}^{b}\delta t)$. See the main texts.

Here ${\hat{U}}^{†a}\equiv \langle \chi_{l}|\phi_{m}^{a}\rangle$ transforms the (a)-frame eigenstate back to the one in the field-free frame. Next, ${\hat{U}}^{b}\equiv \langle \phi_{m}^{b}|\chi_{l}\rangle$ transforms the state defined in the field-free frame to the one in the (b) frame. The time-evolution is operated easily in the (b) frame by adding the phase-shift to the eigenstates defined in the (b) frame, which is performed by multiplying a diagonal matrix $\exp(-i{\hat{\epsilon }}^{b}\delta t)$. Figure 1 presents a schematic diagram of the numerical method.

We emphasize again that all the operations are strictly unitary in this time evolution if the eigenvalue problems are solved correctly. Hence, it is free from any numerical errors generated in the temporal propagation. Instead, the temporally-discretized Hamiltonian is solely responsible for some possible deviations from the correct solution. The discretized Hamiltonian approaches a real Hamiltonian by reducing the step size. The reliable maximum step size depends on the temporal shape of the Hamiltonian rather than the wave function condition. In this method, most of the numerical tasks involve calculating the eigenstates and eigenvalues of the Hamiltonians in every discretized step, which can be conducted using the available built-in functions in various programming languages. A similar numerical approach is used in the Lanczos propagator [34], but with a set of quasi-eigenvalues and quasi-eigenstates defined in a reduced subspace rather than exact eigenvalues and eigenstates in the full Hilbert space. For example, if the exact TISE calculation is numerically demanding, the Lanczos approach can be used with a trade-off between the numerical accuracy and the computing cost.

### 2.2. TDUT in the Strong-Field Ionization Regime

When a target material ionized by an external laser field is considered, a set of time-independent field-dressed eigenstates of the material at a sepecific time will form a continuum state that is not localized in real space [22]. As a result, the TDUT approach introduced in Section 2.1 is not directly applicable in the strong-field regime because the method requires a set of field-dressed eigenstates in every temporal step. On the other hand, a set of strong-field-dressed eigenstates and eigenenergies can be obtained in the moving frame, so-called the Kramers-Henneberger frame.

In the KH frame [32], the laser-field-dressed Hamiltonian of an atom or a molecule is given by

(5) $$\begin{array}{c} H=\frac{{\vec{p}}^{2}}{2}+V\left(\vec{r}+\vec{\alpha }\left(t\right)\right), \end{array}$$

where $V(\vec{r})$ is the binding potential and $\vec{\alpha }\left(t\right)\equiv \int_{-\infty }^{t}\vec{A}\left(t\right)dt$ indicates the classical trajectory of a free electron exposed to the laser field $\vec{E}\left(t\right)\equiv -\frac{d\vec{A}\left(t\right)}{dt}$. In this moving frame, the eigenstates of a field-dressed Hamiltonian are equivalent to the field-free eigenstates except that they are displaced uniformly from the origin by $-\vec{\alpha }\left(t\right)$. Once the field-free eigenstates of an atom or a molecule are accurately obtained, we can apply the method given in Equation (4).

**Figure 2:**
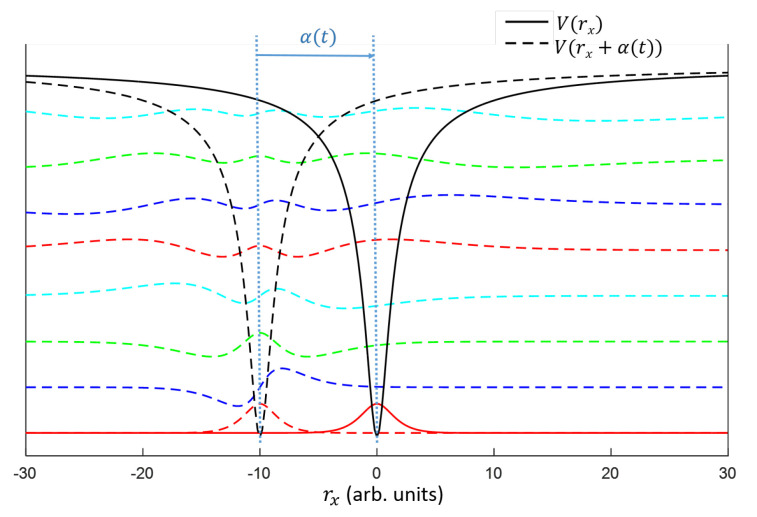
Representation of two frames converted by a translation operator $\hat{U}\left(\vec{\alpha }\left(t\right)\right)$. The black-solid and black-dashed lines are the atomic potentials in the field-free and the field-dressed KH frames, respectively, while the solid red line is the ground state of the atom, and the other colored-dashed lines are eigenstates of the field-dressed KH Hamiltonian Equation (5). The initial ground state is expanded by the eigenstates of the field-dressed Hamiltonian by multiplying $\hat{U}\left(\vec{\alpha }\left(t\right)\right)$ to the initial ground state.

The wave function ψ(t) can be described in terms of the discrete level expansion as Equation (2). Hence, the wave function ψ(t) can be represented by the vector $\hat{\psi }\left(t\right)={\left(a_{1},a_{2},\ldots ,a_{n_{\max}}\right)}^{T}$, e.g., the initial ground state is ${\left(1,0,\ldots ,0\right)}^{T}$. In the moving (laser-field-dressed) frame, $\hat{\psi }\left(t\right)$ is replaced with $\hat{U}\left(\vec{\alpha }\left(t\right)\right)\cdot \hat{\psi }\left(t\right)$, where the matrix $\hat{U}\left(\vec{\alpha }\left(t\right)\right)$ is a translation operator (Figure 2):

(6) $$\begin{array}{c} \hat{U}{\left(\vec{\alpha }\left(t\right)\right)}_{m,n}\equiv \int_{-\infty }^{\infty }\phi_{m}^{*}\left(\vec{r}+\vec{\alpha }\left(t\right)\right)\phi_{n}\left(\vec{r}\right)dr. \end{array}$$

The time evolution of the wave function for a time step dt can be expressed as

(7) $$\begin{array}{c} \hat{U}\left(\vec{\alpha }\left(t\right)\right)\cdot \hat{\psi }\left(t+dt\right)=\exp(-i\hat{\epsilon }dt)\cdot \hat{U}\left(\vec{\alpha }\left(t\right)\right)\cdot \hat{\psi }\left(t\right). \end{array}$$

Here, $\hat{\epsilon }$ is the matrix whose diagonal elements are eigenenergies of the field-free eigenstates. To rewrite the wave function in Equation (7) in the field-free frame, the transpose of $\hat{U}\left(\vec{\alpha }\left(t\right)\right)$ needs to be multiplied, resulting in

(8) $$\begin{array}{c} \hat{\psi }\left(t+dt\right)={\hat{U}}^{T}\left(\vec{\alpha }\left(t\right)\right)\cdot \exp(-i\hat{\epsilon }dt)\cdot \hat{U}\left(\vec{\alpha }\left(t\right)\right)\cdot \hat{\psi }\left(t\right). \end{array}$$

Although the translation operator $\hat{U}\left(\vec{\alpha }\left(t\right)\right)$ is a unitary matrix in principle, in case of the numerical calculation, the unitarity of $\hat{U}\left(\vec{\alpha }\left(t\right)\right)$ is not ensured. Furthermore, the matrix becomes less unitary as the spatial displacement $\vec{\alpha }\left(t\right)$ increases. The matrix $\hat{U}\left(\vec{\alpha }\left(t\right)\right)$ corresponds to the unity matrix when $\vec{\alpha }\left(t\right)$ is zero. For other cases, because $\hat{U}\left(\vec{\alpha }\left(t\right)\right)$ is responsible for transforming a wave function in the $\vec{r}$ frame into that in the $\vec{r}+\vec{\alpha }\left(t\right)$ frame, if $\vec{\alpha }\left(t\right)$ is too large, the initial wave function will be placed outside of the spatial boundary so that $\hat{U}\left(\vec{\alpha }\left(t\right)\right)$ is no longer unitary.

We have identified that a spatial displacement of 0.2, which is the size of spatial grid used in the numerical calculations, does not ensure that the matrix is unitary, i.e., $det|\hat{U}\left(\alpha_{x}\left(t\right)=0.2\right)|<1$. To obtain a unitary translation operator $\hat{U}\left(\delta x\right)$, the matrix elements were calculated in the momentum domain by

(9) $$\begin{array}{c} \hat{U}{\left(\delta x\right)}_{m,n}\equiv \int_{-\infty }^{\infty }\exp(ip_{x}\delta x){\tilde{\phi }}_{m}^{*}\left(\vec{p}\right){\tilde{\phi }}_{n}\left(\vec{p}\right)dp, \end{array}$$

where ${\tilde{\phi }}_{n}\left(\vec{p}\right)$ is the Fourier-transformed, normalized wave function.

In the momentum domain, we have calculated the unit translation operator $\hat{U}\left(\delta x\right)$ with $\delta x=10^{-4}$. This operator is quasi-unitary, satisfying $|\hat{U}\left(\delta x\right)|∼1$ with a possible deviation of only $10^{-15}$. When the laser field is linearly polarized in the *x* direction and $\vec{\alpha }\left(t\right)\equiv \alpha_{x}\left(t\right)\equiv \alpha \left(t\right)$, an arbitrary translation operator $\hat{U}\left(\alpha \left(t\right)\right)$ can be obtained from the multiplication of the unit displacement operator, i.e., $\hat{U}\left(\alpha \left(t\right)\right)\equiv \hat{U}{\left(\delta x\right)}^{\alpha (t)/\delta x}$. By applying this operator $\hat{U}\left(\delta x\right)$, the TDSE can be solved by the following procedure.

For the time evolution of the wave function, first we expand the wave function $\hat{\psi }\left(t\right)$ with respect to the field-dressed eigenstates, which are the field-free eigenstates spatially displaced by α(t) from the origin. This expansion is conducted by multiplying the quasi-unitary matrix $\hat{U}{\left(\delta x\right)}^{\alpha (t)/\delta x}$ to the wave function $\hat{\psi }\left(t\right)$. The negative unit translation operator $\hat{U}{\left(\delta x\right)}^{-1}$ is, therefore, defined as $\hat{U}{\left(\delta x\right)}^{T}$. Afterwards, the expanded wave function $\hat{U}{\left(\delta x\right)}^{\alpha (t)/\delta x}\cdot \hat{\psi }\left(t\right)$ is multiplied by the diagonal matrix $\exp(-i\hat{\epsilon }dt)$, resulting in phase shifts of the expanded eigenstates. Thereafter, we recover the wave function described from the spatially displaced (field-dressed) frame to the field-free frame. This is conducted by multiplying the transpose of $\hat{U}{\left(\delta x\right)}^{\alpha (t)/\delta x}$. By repeating the procedure for the overall time evolution, we can express the full time-evolution operator ${\hat{U}}_{\mathrm{total}}$ given by

(10) $$\begin{array}{c} {\hat{U}}_{\mathrm{total}}=\left[\prod_{l=0}^{l_{f}}\hat{U}{\left(\delta x\right)}^{-\alpha (t_{l})/\delta x}\cdot \exp(-i\hat{\epsilon }dt)\cdot \hat{U}{\left(\delta x\right)}^{\alpha (t_{l})/\delta x}\right], \end{array}$$

where $t_{l}\equiv t_{0}+ldt$, with *l* being an integer. $l_{f}$ is defined as $t_{f}=t_{0}+l_{f}dt$.

By combining the relation α(t+dt)−α(t)=A(t)dt with a temporal boundary condition $A\left(t_{0}\right)=A\left(t_{f}\right)=0$, the expression for ${\hat{U}}_{\mathrm{total}}$ can be further simplified to

(11) $$\begin{array}{c} {\hat{U}}_{\mathrm{total}}=\left[\prod_{l=0}^{l_{f}}\exp(-i\hat{\epsilon }dt)\cdot \vec{U}{\left(\delta x\right)}^{A(t_{l})dt/\delta x}\right]. \end{array}$$

In Equation (11), the number of multiplications $A(t_{l})dt/\delta x$ for each time step *l* is rounded. The numerical error caused by the rounding is ignorable by setting δx at ∼$10^{-4}$, far smaller than the spatial grid size of 0.2. We use a set of eigenstates of the field-free Hamiltonian to calculate the time evolution operator. To obtain the field-free eigenstates by solving a Hermitian TISE, we considered a reflection boundary rather than an absorbing or transparent one. In the absorbing and transparent boundaries, the Hamiltonians are non-Hermitian, so that the numerical solutions of the TISE require additional techniques [22]. The resulting high-lying continuum states in the reflection boundary are, therefore, well confined inside the boundary, indicating that the full propagator Equation (11) intrinsically includes unphysical reflections of the wave function at the boundary. To avoid such unphysical reflections, an absorbing boundary matrix $\hat{W}$ is multiplied to the wave function at every time step. The matrix elements are described as

(12) $$\begin{array}{c} {\hat{W}}_{m,n}\equiv \int_{-\infty }^{\infty }\phi_{m}^{*}\left(x\right)W\left(x\right)\phi_{n}\left(x\right)dx, \end{array}$$

where W(x) is a unity function that smoothly decays to zero near the boundary. We can express the full time-evolution propagator, including the absorbing boundary, as

(13) $$\begin{array}{c} {\hat{U}}_{\mathrm{total}}=\left[\prod_{l=0}^{l_{f}}\hat{W}\cdot \exp(-i\hat{\epsilon }dt)\cdot \hat{U}{\left(\delta x\right)}^{A(t_{l})dt/\delta x}\right]. \end{array}$$

An initial state converts to the corresponding final state by multiplying the operator Equation (13).

## 3. Results of the Simulation

**Figure 3:**
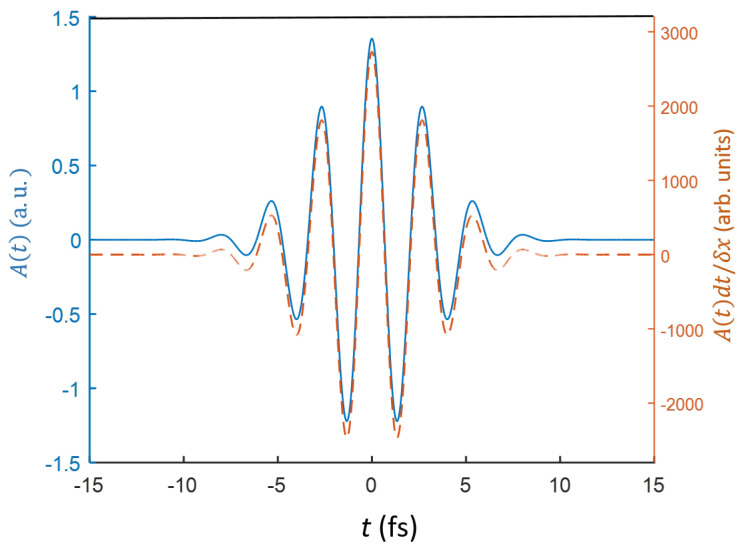
Temporal profiles of the vector potential A(t) and the function A(t)dt/δx, which is rounded.

The numerical method shown in Section 2 was tested with a one-dimensional soft-core potential $V\left(x\right)=-\frac{1}{\sqrt{x^{2}+1}}$. The atom was irradiated with a pulsed laser field,

(14) $$\begin{array}{c} E\left(t\right)=E_{0}\exp\left[-2\ln2\left(t^{2}/\tau^{2}\right)\right]\sin\left(\omega t\right), \end{array}$$

where $E_{0}$ is the peak strength of the laser field, and τ is the FWHM (fixed at 5 fs). The central frequency of the laser ω was set to λ≡2πc/ω=800 nm, where *c* is the speed of light. Figure 3 presents the temporal profiles of the vector potential, $A\left(t\right)\equiv -\int_{-\infty }^{t}E(t)dt$, and the function, A(t)dt/δx, used to define the translation operator, $\hat{U}{\left(\delta x\right)}^{A(t_{l})dt/\delta x}$ as included in Equation (11). dt was set to 0.2 in the calculations. We tested the dt dependence of the numerical solution by varying its values from 0.5 to 0.01 (not shown). The numerical solution converged well for all the values.

To obtain the eigenstates and eigenvalues of the atom, the TISE was solved. The one-dimensional *x* space, whose reflection boundaries were set at −614.4 and 614.2, was employed. The space was discretized by 6144 grids, each with a size of 0.2. There were 43 bound states with negative energies and 6101 continuum states with positive energies. The ionization energy of the atom, i.e., the eigenenergy of the ground state, was obtained as −18.23 eV. We set $n_{\max}$, the number of the lowest-lying states, to 50∼2000. The number of the necessary states depends on the peak laser intensity.

**Figure 4:**
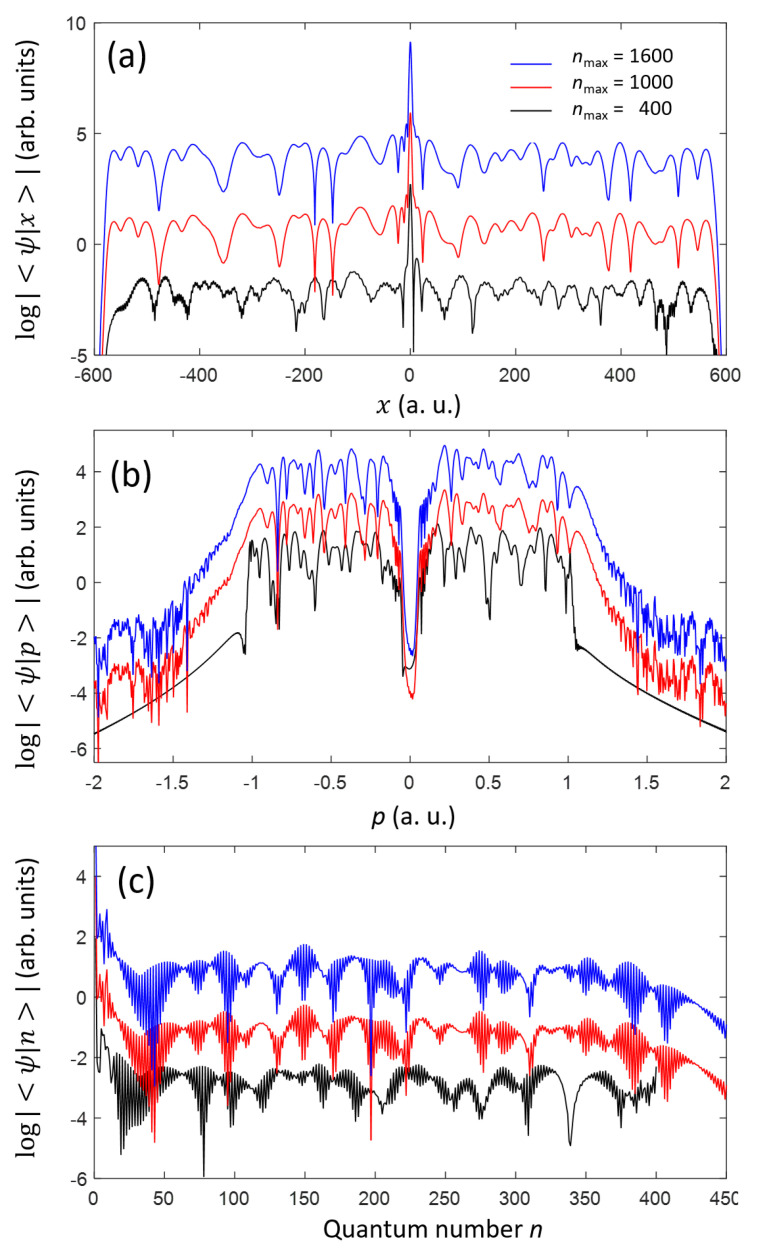
Atomic wave function irradiated with a short infrared laser pulse described by Equation (14). The ground state is used as an initial state. The wave function at $t=t_{f}=$ 15 fs is shown in the space (**a**) and momentum (**b**) domains, and also by the quantum number *n* representation (**c**). For the plots in the momentum domain (**b**), only the continuum states are considered. The lines are displaced vertically for visual convenience.

Figure 4 shows the final wave function after a laser pulse, described by Equation (14), has passed. The peak intensity of the pulse is $2.0\times 10^{14}\;W/{\mathrm{cm}}^{2}$. We show the results obtained when the numbers of discrete states $n_{\max}$ are 400, 1000, and 1600. For the above laser condition, the results obtained using $n_{\max}=$ 1000 and 1600 do not show any noticeable variations in the all represented domains: space (Figure 4a), momentum (Figure 4b), and quantum number (Figure 4c). The agreement between the results for $n_{\max}$ = 1000 and 1600 indicates that the TDUT method converges by using $n_{\max}>1000$ discrete states, as the basis set. When $n_{\max}$ is set to 400, the results from the TDUT show clear deviations from the other results. The deviations are observed not only in the continuum states (n> 44), but also in the bound states (n≤ 43), meaning that a noticeable amount of field-ionized electrons can be recaptured to the bound states.

When the applied laser field is stronger and the excitation of the higher-lying states becomes essential, more discrete states are required for the TDUT calculation. We have evaluated, therefore, this quantity as a function of the peak laser intensity. For a given peak intensity condition and from the initial ground state, we have recalculated the final states until a converged result is obtained by increasing $n_{\max}$ by every 50. The convergence is evaluated from the parameter,

(15) $$\begin{array}{c} \delta O\left(n_{\max}\right)\equiv |U_{\mathrm{total}}\left(n_{\max}\right)|1>-U_{\mathrm{total}}\left(n_{\max}+50\right){\left|1>\right|}^{2}, \end{array}$$

where |1> represents the ground state. By increasing $n_{\max}$, the final wave function $U_{\mathrm{total}}\left(n_{\max}\right)|1>$ must converge to a state so that $\delta O(n_{\max})$ becomes negligibly small. When $\delta O(n_{\max})$ is smaller than $2.5^{-7}$, $n_{\max}$ is selected as $n_{\mathrm{cutoff}}$ and the convergence test is terminated.

**Figure 5:**
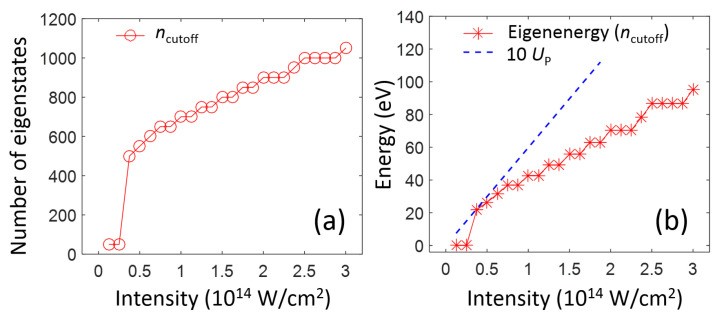
$n_{\mathrm{cutoff}}$ (**a**) and the eigenenergy at $n_{\mathrm{cutoff}}$ (**b**) as functions of the peak laser intensity. See the main text.

In Figure 5a, $n_{\mathrm{cutoff}}$ is shown as a function of the peak laser intensity. For lower intensities, such as 0.125–0.25 $\times \;10^{14}\;W/{\mathrm{cm}}^{2}$, the TDUT method provides converged results by using less than 50 discrete states. In this case, the atomic response can be described by perturbation theory, considering only a few discrete low-lying bound states. When the peak laser intensity is high, the dynamics is governed by tunneling ionization. In Figure 5a, the dramatic increase in $n_{\mathrm{cutoff}}$ at an intensity of $0.375\times 10^{14}\;W/{\mathrm{cm}}^{2}$ is observed, showing the transition from the perturbative to the tunneling regimes. After this transition point, $n_{\mathrm{cutoff}}$ continues to increase with the increasing peak laser intensity. In Figure 5b, the eigenenergy of a discrete state, whose quantum number corresponds to $n_{\mathrm{cutoff}}$ is plotted as a function of the peak laser intensity. Additionally, 10 $U_{P}$, where $U_{P}$ is the laser-intensity-dependent ponderomotive energy, is represented by the blue dashed line, and 10 $U_{P}$ is a maximum possible energy of an electron, which is generated after being elastically rescattered by the atomic core in the above-threshold ionization (ATI) dynamics [7]. The number of states needed for the TDUT method can be approximately determined by the maximum electron energy, because much higher-energy states cannot physically exist. The necessary number of states in the calculation will depend on the dynamics to be investigated, because such high-energy states have ignorable influence on the bound electron dynamics. In fact, it has been clarified that some of strong-field-ionized electrons having low kinetic energy can survive as Rydberg states [9,15,16]. To study only the bound-state dynamics, which is possibly coupled with the strong-field ionization, the number of included states can be further reduced.

**Figure 6:**
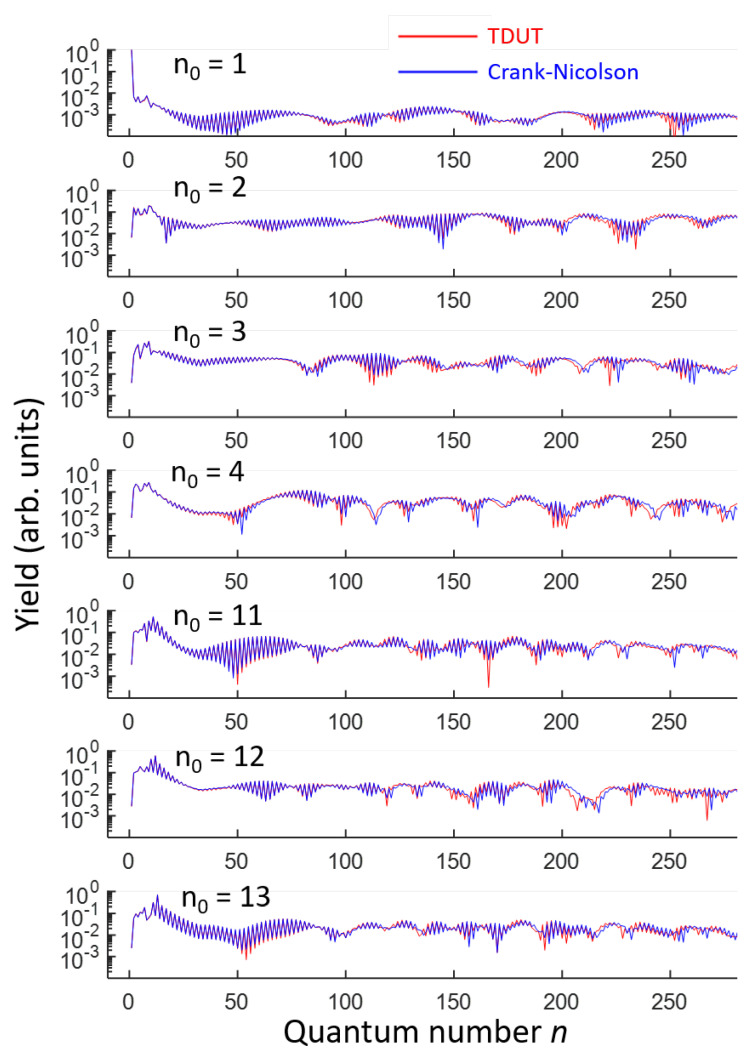
Final states in the quantum number representation for several different initial wave functions, numbered by $n_{0}=1,2,3,4,11,12$, and 13, shown in the discrete state representation.

We are able to apply the full time-evolution operator Equation (13) to any initial wave function. Figure 6 shows the final states obtained by multiplying a full-time evolution operator to several different initial bound states ($n_{0}=$ 1, 2, 3, 4, 11, 12, and 13), where $n_{0}$ is the quantum number of the initial state. The temporal shape of the laser is the same as Equation (14), and the peak laser intensity is set at $1.0\times 10^{14}\;W/{\mathrm{cm}}^{2}$. The time evolution operator is calculated with $n_{\max}=2000$. The results obtained by the Crank–Nicolson method are also shown in Figure 6.

For the initial ground state $n_{0}=1$, the ground state population remains almost equal to 1, indicating that the depletion by tunneling ionization is not so significant. Other initial low-lying bound states, i.e., when $n_{0}=2,3$, and 4, whose eigenenergy values are −7.49, −4.13, and −2.53 eV, respectively, result in significant depletion by tunneling. However, for the Rydberg bound states $n_{0}=11,12$, and 13, with the eigenenergy values of −0.40, −0.34, and −0.29 eV, respectively, the depletion of the initial states is less dominant than that in the lower-lying bound states. This is due to the stabilization of Rydberg states [36,37]. For the defined initial conditions, the final quantum state distributions obtained by both methods exhibit some visible deviations, due to the difference in the absorbing boundary conditions, in the continuum states (n>43). The absorbing boundary function W(x), defined in the space domain for the Crank–Nicolson method, has been transformed by the matrix ${\hat{W}}_{m,n}$ of the discrete state representation given by Equation (12). For the bound states (n≤43), which are not directly affected by the absorbing boundary conditions, the numerical results from both the methods are consistent.

## 4. Summary and Outlook

In this paper, we have introduced a numerical method based on the time-dependent unitary transformation (TDUT). This approach, first demonstrated for molecular rotational dynamics in Refs. [29,30], is implemented to the strong-field-ionization regime of an atom or a molecule. In the Kramers-Henneberger frame, the field-dressed eigenstates are identical to the field-free eigenstates excluding their spatial displacement, which becomes a useful advantage to calculate the unitary operators to propagate the electronic wave function in every temporal step. In the TDUT method, matrices and vectors associated with an atom, such as eigenstates, eigenenergies, and a unit translation operator $\hat{U}\left(\delta x\right)$ (Equation (9)), need to be calculated in advance (the calculation took 5 min for $n_{\max}=2000$). The matrices and vectors can be reapplied under different laser conditions. For the same reason, the method will be even more beneficial in a very long-pulsed case. A final bound state population after pico- to nanosecond laser pulse irradiation can be calculated. In the long pulsed regime, it becomes more important to remove any unphysical reflections at the boundaries, which has been done by multiplying an absorbing boundary matrix (Equation (12)) at every temporal step. In the present work, after the matrix elements were prepared, it took approximately 4 s and 20 s with the TDUT and Crank-Nicolson methods, respectively, by using a personal computer (Intel(R) Core(TM) i9-9900K CPU, 128 GB RAM, Windows 10). This method can be a useful tool in calculating and analyzing bound electron dynamics coupled with strong-field ionization, not only in the one-dimensional system, but also in three-dimensional molecular systems.

## Data Availability

The data presented in this study are available on request from the corresponding author. The data are not publicly available due to ethical restriction.

## Abbreviations

The following abbreviations are used in this manuscript:

TISE	Time-independent Schrödinger equation
TDSE	Time-dependent Schrödinger equation
KH	Kramers-Henneberger
SFA	Strong-field approximation
FTI	Frustrated tunneling ionization
ATI	Above-threshold ionization
HHG	High-harmonic generation
TDUT	Time-dependent unitary transformation
FWHM	Full width at half maximum
